# Supplementary material for: Markovian language model of the DNA and its information content
Source: R Soc Open Sci. 2016 Jan 6;3(1):150527. doi: 10.1098/rsos.150527 (PMC4736934; doi:10.1098/rsos.150527)
Supplement: Supplementary Materials for the details of methods [file rsos150527supp1.pdf]

Supplementary material

1 Determination of word length

The topological entropy of the DNA is defined as

$$H_T = \lim_{\mathbf{2L}\to\infty} \frac{log(\mathbf{N_{2L}})}{\mathbf{2L}}, \tag{1}$$

where N_{2L} denotes the number of different words of length $2L$ appearing anywhere in the symbolic sequences. Naturally, in practice, we drop the limit and estimate H_T to be the one such that it remains roughly invariant as L is changed within a finite interval. We also define the Shannon entropy rate

$$S_T = -\sum_{i}^{N_{2L}} \frac{p_i\ log(p_i)}{\mathbf{2L}} \tag{2}$$

where, p_i is the probability of words of length $2L$ appearing anywhere in the symbolic sequence.

(a) **(b)**

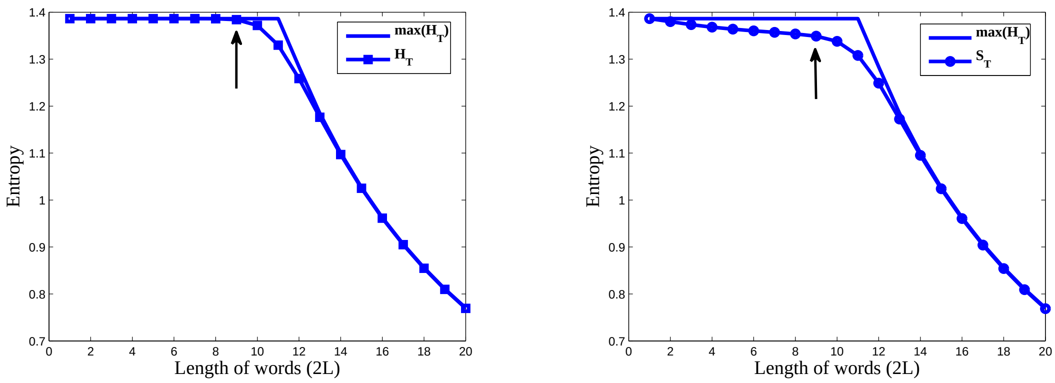

Figure 1: Different entropy measure for determining the length of words, where horizontal axis shows different size for words. (a) Shows the topological entropy (H_T) represented by squares and max(H_T) drawn by the full line. (b) Shows the Shannon entropy rate (S_T) represented by circles and max(H_T) by the full line.

Fig. 2(a) displays the topological entropy and the upper bound of the topological entropy as a function of $2L$. If any sequence of length $2L$ can be seen in the DNA then $H_T = \log(4)$, a constant value. There are however, two conditions on which max$(H_T) < log(4)$. First, if there are not enough nucleotides in the DNA to form all the sequences, and second, if there are forbidden sequences. Therefore, we define the max(H_T) as log(4) if the DNA is sufficiently long and all sequences are observed $(4,600,000 - 2L > 4^{2L})$ or

$$max(H_T) = \frac{1}{2L}\log(4,600,000 - 2L). \tag{3}$$

Assuming ($N_{2L} = 4,600,000 - 2L$) if the DNA length is not long enough to allow that any sequence of length L appears at least once, resulting in that $H_T \leq max(H_T)$. Fig. 2(b) displays the Shannon entropy S_T, and the upper bound on topological entropy. The similarity in the results for H_T and S_T suggests that the optimal word length to choose for creating the words of the genome must be 8. Therefore, we chose the size of length $2L$ words to be 8. We obtain that $H_T(L = 4) \cong 1.38$, which is in fact close to the largest possible topological entropy that sequences compromising of a finite alphabet of 4 symbols can achieve, i.e., log (4). That suggests the DNA has a large stochastic component, in terms of likelihood of finding different words. H_T always decays for larger L and therefore, the only roughly invariant plateau is for $2L \leq 8$.

2 Recurrence of the group of words in the DNA

It is not the scope of this paper to study deeply the recurrence of words in our Markovian model of the DNA. But, we want to explore the advantageous implications of our model by showing how recurrence can be obtained from the quantities already defined. In addition, the statistics of returns is a powerful tool to characterize the DNA. We consider the probability density of first Poincaré returns (FPR) [1], from which we can specify the likelihood that a word in a group will be followed by a word belonging t same group that is τ nucleotides ahead in the DNA sequence. Let a group of words w_i represent points in a box i in an order-T partition with probability $p(i)$, and let $p^F_\tau(i|i)$ to represent the transition probability for a sub-group of words in w_i that encode points that after τ iterations fall again for the first time in the initial box i. The density of first Poincaré returns of length T of box S_i would then be calculated by $\rho(i, \tau) = p^F(i|i)_\tau$. Assuming box i to be off diagonal it is to be expected that $p(i)p(i|i)_T$ represents the probability density of first returns of words in box i, since $p(i|j)_T = p(i|j)^F_\tau$. Take as an example the points representing sequences that contains the sub-sequences "23.122312" in an order-4 partition. These points are in off-diagonal box name '23.12'. All points whose symbolic sequences have '23.122312...' will be iterated off the box '23.12' and only firstly return after 4 nucleotides shifts (iterations). To simplify the result even further, we notice that the correlation decays to approximately zero at $\tau = T$, then $p(i|i)_T \cong p(i)$. So, the density of shortest first returns in box i for observing a group of words w_i repeating after T nucleotides for these special boxes could then be estimated by

$$\rho(i, \tau = T) = p^F(i|i)_T = p(i). \tag{4}$$

The recurrence measures the probability density of observing two equal words w_i separated by a distance of T nucleotides. From the Eq (4), we can clearly see a Markov property of our model. To calculate returns of any T length all that is needed is the knowledge of $p(i)$. Therefore, the "piece" $p(i)$ can describe the whole, the density of first returns for the group of words w_i.

3 Markov process, mixing, and correlation

Markov process, systems or chain are said to be memoryless in nature because one can predict the future solely based on its present state [2]. Let $\mathbf{P}$ be the transition matrix of a Markov chain, and $\mathbf{u}$ be the probability vector which represents the initial arbitrary vector. Then, the probability that the states of the chain are after n iterations is given by

$$\mathbf{u}^{(n)} = \mathbf{u}\mathbf{P}^n. \tag{5}$$

When $\mathbf{P}$ has $\sum_i \mathbf{P}_{ij} = 1$ and has only 1 positive eigenvalue equal to 1, then $\mathbf{P}$ describes a Markov chain whose final state probabilities $\mathbf{u}^{(n)}$ will be independent of the initial distribution $\mathbf{u}$. $\mathbf{u}^{(n)}$ are solely given by the eigenvalues of $\mathbf{P}$ and mostly important $\mathbf{P}^n_{ij} = \mathbf{u}^{(n)}_j$. To construct our approximate markov model, we check for the existence of weak mixing in the DNA.

$$C(N, \tau) = \sum_{i,j} [p(i)_N p(i|j)_\tau - p(i)p(j)] = 0. \tag{6}$$

Remind that $p(i|j)_\tau$ represents the transition probability of points moving from box i to box j after τ iteration. If Eq. (7) is satisfied for all i and j, then

$$p(i)p(j) = p(i)p(i|j)_\tau, \tag{7}$$

A condition for a strong mixing, and then Eq. (6) would also be satisfied. Assume now that Eq. (7) is verified for all i and j. Let us now prove that this implies in a memoryless Markov chain. It is always true that

$$p(j) = \sum_i p(i)p(i|j)_\tau \tag{8}$$

Rewriting this equation in matrix form, we arrive at

$$\vec{P} = \vec{P}\mathbf{P}(i|j)_\tau \tag{9}$$

If $p(j) = p(i|j)_\tau$ [Eq. (7)] is satisfied for all i and j, then the only possibility for Eq. (9) to be satisfied is when $\mathbf{P}(i|j)_\tau$ is equal to

$$\mathbf{P}(i|j)_\tau = \vec{P}\begin{pmatrix} p(1|1)_\tau & p(1|2)_\tau & \cdots & p(1|N)_\tau \\ p(1|1)_\tau & p(1|2)_\tau & \cdots & p(1|N)_\tau \\ \vdots & \vdots & \ddots & \vdots \\ p(1|1)_\tau & p(1|2)_\tau & \cdots & p(1|N)_\tau \end{pmatrix}$$

where, $\mathbf{P}(i|j)_\tau$ is the transition matrix with elements i and j. p(1/1) represents the transition probability of points moving from box i to box j and $\vec{P} = (p(1), p(2), \ldots, p(N))$ is a probability vector representing initial arbitrary vector. For example if we consider $j = 2$, then,

$[\vec{P}\mathbf{P}(i|j)_\tau]_2 = P(2) = p(1)p(1/2) + p(2)p(1/2) + p(3)p(1/2) + \ldots + p(N)p(1/2) = p(1/2)\sum p(i) = p(1|2)$.

Therefore, $p(j) = p(i|j)_\tau$.
To construct our model, we do not verify that $p(i|j)_\tau = p(j)$. The Markovian property obtained in this work is constructed in a sense of Eq. (6), which defines weak mixing.
A Markov chain can be associated to a Markov partition. The probabilities of finding points on the order-T partition $p_N(i)$ and the transition probabilities $p_N(i|j)_\tau$ would then be provided by the partitions. There is an extra requirement for the partition to be Markov:

i) the union of intervals $\equiv$ *domain*

ii) No overlapping of unions of higher order partition with lower order partitions.

It is clear that the order-2 and order-4 partition considered in this work satisfy condition i) and ii). So, the partition provides a mixing system where cells behave as Markov

4 Relationship between symbolic and integer box names

The sides of a partition box is equal to 4^{-L_n}, and the coordinates in the symbolic space of a box defined by a past symbolic sequence $s_{-L_n}s_{-L_n+1}\ldots s_{-1} \,.\, s_1 \ldots s_{L_n-1}s_{L_n}$ is given by the real numbers obtained from the symbolic sequence: $[\frac{s_{-1}4^{L_n-1}}{4^{L_n}-1}, \frac{s_{-1}4^{L_n-1}+4^{-L_n}}{4^{L_n}-1}[$ for the past δ coordinate and $[\frac{s_1 4^{L-1}}{4^{L_n}-1}, \frac{s_1 4^{L-1}+4^{-L_n}}{4^{L_n}-1}[$ for the future γ coordinate. The integer name of a box can be obtained from the most T significant symbols of the symbolic sequence. For example, assume $L = 4$ and $T = 4$ (the order of partition). Then, a point encoding a sequence of length $2L$ of the DNA $[s_{-L_n}s_{-L_n+1}\ldots s_{-1} \,.\, s_1 \ldots s_{L_n-1}s_L]$ belongs to a box whose symbolic name is $[s_{-2}s_{-1}.s_1s_2]$.
The integer representation of the box name is a value that is used to create the index of the transition matrices given by $[i = s_{-2}4^1 + s_{-1}4^0, j = s_1 4^0 + s_2 4^1]$. In general, the integer index of matrices representing a box of an order-T partition in the symbolic space of a point in position j can be calculated by

$$i = \sum_{i=1}^{\frac{T}{2}} s_{j-i} 4^{\frac{T}{2}-i} \tag{10}$$

and

$$j = \sum_{i=1}^{\frac{T}{2}} s_{j+i-1} 4^{\frac{T}{2}-i} \tag{11}$$

4.1 Comparison of the Markov model of *E. coli* with genes of *Shigella dysenteriae* and *Rhodococcus fascians*

To understand if our model of the *E.coli* can be used to detect similar genes in other organisms, we consider a closely related bacterium to *E coli*, *Shigella dysenteriae.* Many studies suggest these organisms share a similar evolutionary process [3]. As shown in Fig. 1(a), the blue empty squares depicts (S_p, S_n) values for the genes of *S. dysenteriae* and in red colour crosses the same quantities for the *E. coli* genes, where in the calculation of S_p and S_n of both organisms we have compared the adjacency matrix for genes in *S. dysenteriae*($\mathbf{G}^{S.dys}(g_i,t^*)$) and in *R. fascians* ($\mathbf{G}^{R.fas}(g_i,t^*)$) with the adjacency matrix of *E.coli* ($\mathbf{G}^{E.coli}(t^*)$) . The model constructed from group of word of *E. coli* can predict the genes of *S. dysenteriae* at different levels. In more rigorous terms, $TP^{S.dys}(g_i,t^*) = \sum_{ij}^{1,1}(G_{ij}^{E.coli}(t^*) - G_{ij}^{S.dys}(g_i,t^*))$, where we only take into consideration all the i and j values of $G_{ij}^{E.coli}(t^*)$ and $G_{ij}^{S.dys}(g_i,t^*)$ which are equal to 1. The symbol $\sum_{ij}^{1,1}$ represents a summation that is only carried out when the variables inside the argument are equal to the super index. FN is the number of words that were wrongly predicted, $FN^{S.dys}(g_i,t^*) = \sum_{ij}^{1,0}(G_{ij}^{E.coli}(t^*) - G_{ij}^{S.dys}(g_i,t^*))$, this can happen only when $G_{ij}^{E.coli}(t^*) = 1$ and $G_{ij}^{S.dys}(g_i,t^*) = 0$, meaning that a transition from the group of words in box i are mapped to box j are not present but have been wrongly predicted by the model. $TN^{S.dys}(g_i,t^*) = \sum_{ij}^{0,0}(G_{ij}^{E.coli}(t^*) - G_{ij}^{S.dys}(g_i,t^*))$, in this case we consider all the values of $G_{ij}^{E.coli}(t^*)$ and $G_{ij}^{S.dys.}(g_i,t^*)$ that are equal to zero, meaning that the a transition from the group of words in box i are mapped to box j do not exist and the model also does not predicts them. $FP^{S.dys}(g_i,t^*) = \sum_{ij}^{0,1}(G_{ij}^{E.coli}(t^*) - G_{ij}^{S.dys}(g_i,t^*))$, happens when $G_{ij}^{E.coli}(t^*) = 0$ but $G_{ij}^{S.dys}(g_i,t^*) = 1$. $S_p(t^*)$ is very small for any t^* and for small t^* also $S_n(t^*)$ is close to 1. In addition, the closeness of the points indicate that genes in these two bacteria share remarkable similarities. That is to be expected since they share a similar evolutionary relationship. On the other hand, when we compare the S_n and S_p space of the *E.coli* and *Rhodococcus fascians* (in Fig. 7(b)), a bacterium that does share any similarity with *E. coli*, we notice that predictability can be high however the differences in S_p and S_n indicate differences in the genes of these organisms. Similarly, $TP^{R.fas}(g_i,t^*) = \sum_{ij}^{1,1}(G_{ij}^{E.coli}(t^*) - G_{ij}^{R.fas}(g_i,t^*))$, where we only take into consideration all the i and j values of $G_{ij}^{E.coli}(t^*)$ and $G_{ij}^{R.fas}(g_i,t^*)$ which are equal to 1. The symbol $\sum_{ij}^{1,1}$ represents a summation that is only carried out when the variables inside the argument are equal to the super index. FN is the number of words that were wrongly predicted, $FN^{R.fas}(g_i,t^*) = \sum_{ij}^{1,0}(G_{ij}^{E.coli}(t^*) - G_{ij}^{R.fas}(g_i,t^*))$, this can happen only when $G_{ij}^{E.coli}(t^*) = 1$ and $G_{ij}^{R.fas}(g_i,t^*) = 0$, meaning that a transition from the group of words in box i are mapped to box j are not present but have been wrongly predicted by the model. $TN^{R.fas}(g_i,t^*) = \sum_{ij}^{0,0}(G_{ij}^{E.coli}(t^*) - G_{ij}^{R.fas}(g_i,t^*))$, in this case we consider all the values of $G_{ij}^{E.coli}(t^*)$ and $G_{ij}^{R.fas}(g_i,t^*)$ that are equal to zero, meaning that the a transition from the group of words in box i are mapped to box j do not exist and the model also does not predicts them. $FP^{R.fas}(g_i,t^*) = \sum_{ij}^{0,1}(G_{ij}^{E.coli}(t^*) - G_{ij}^{R.fas}(g_i,t^*))$, happens when $G_{ij}^{E.coli}(t^*) = 0$ but $G_{ij}^{R.fas}(g_i,t^*) = 1$. From the BLAST2 results of ncbi a 0% similarity was found between these two bacteria, suggesting that they are highly diverse when it comes to global alignment search. There are many methods to study the sequence similarity but we use the most popular approach to study search similarity within the genes, the “Basic Local Alignment Search Tool” (BLAST) [4, 5]. It calculates an “expect value” that estimates how many matches occur at a given score which gives an score to the aligned sequences. For our analysis, we use BLAST2, that helps in aligning any 2 sequences and gives a score for them.

The closeness of points in the (S_p,S_n) space can be used to identify genetic similarities (and dissimilarities) between *E. coli* and these two other organisms. Figure 3 is an amplification for $N = 256$ and $t^* = 0$ where we show how the (S_p,S_n) value for *E.coli* overlaps with these values for

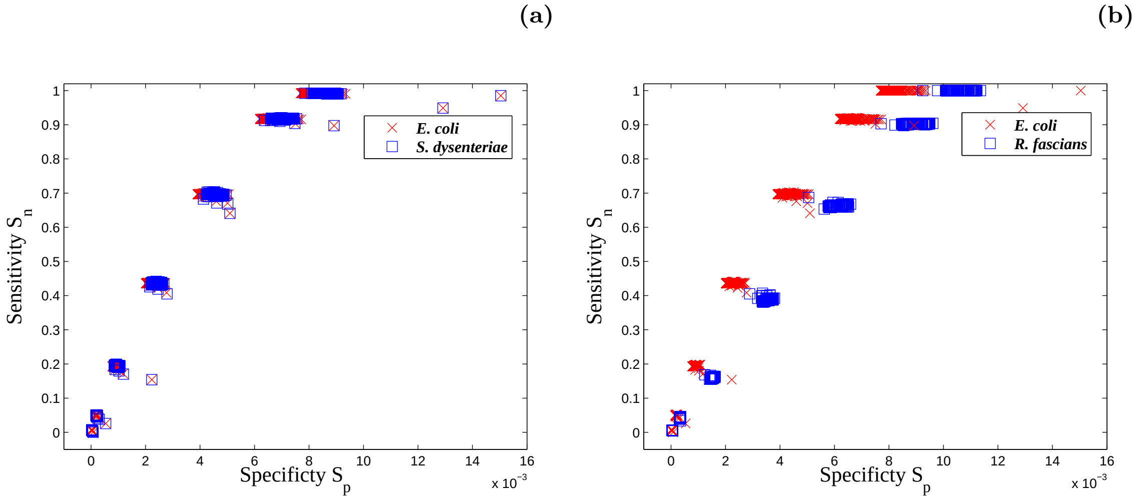

Figure 2: Prediction of genes of (a) *Shigella dysenteriae*, shown with blue empty squares with the Markov model of *Escherchia coli* represented by red crosses , (b) *Rhodococcus fascians* represented by blue empty squares with the Markov model of *Escherchia coli* represented by red crosses. Horizontal axis shows Specificty S_p and vertical shows sensitivity S_n. From bottom to the top 7 different clusters represent results respectively from ($\tilde{N}_V = 5$, $t^* = 0.30$), ($\tilde{N}_V = 27$, $t^* = 0.27$), ($\tilde{N}_V = 85$, $t^* = 0.25$), ($\tilde{N}_V = 153$, $t^* = 0.23$), ($\tilde{N}_V = 207$, $t^* = 0.20$), ($\tilde{N}_V = 241$, $t^* = 0.15$) and ($\tilde{N}_V = 256$, $t^* = 0.0$).

the *S. dysenteriae*, showing similarity among the genes responsible for these values. The following genes *thrA*, *thrB*, *yaaJ*, etc. are considered by the BLAST2 to be 98% similar. On the other hand, the overlapping between *R. fascians* and *E. coli* happens for just one single gene. When we analysed this gene using BLAST2, the results suggested that it had no significant similarity. Suggesting that *R. fascians* is a plant bacteria and is therefore distant from *E. coli* in terms of evolutionary relationship and genomic build up.

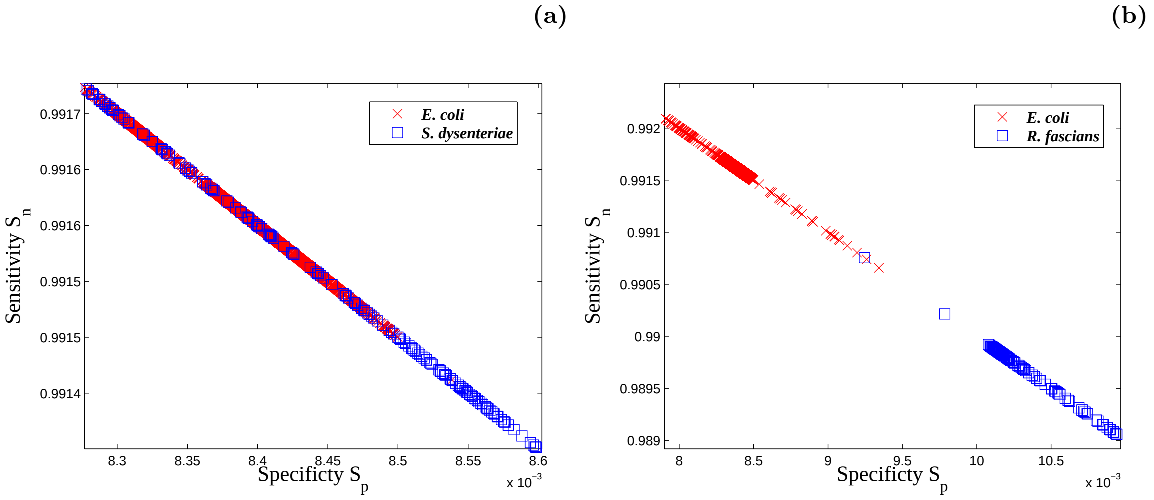

Figure 3: Amplication for (S_p, S_n) $\tilde{N}_V$=256, $t^* = 0$ for genes of *E. coli* represented by red crosses and (a) *Shigella dysenteriae* represented by blue squares, (b)*Rhodococcus fascians* represented by blue squares. Horizontal axis shows Specificty S_p and vertical shows sensitivity S_n.

Despite the fact that (S_p, S_n) values are overlapping, genes might still be symbolic distant. To quantify how distant genes with different (S_p, S_n) value overlapping are, providing a qualitative measure for the similarity, we measure the average minimum distance of the points in the small box in Fig. 8(b). We find that for the mean minimum distance between (S_p, S_n) values from the genes of *E. coli* and *S. dysenteriae* is 5.0095e-04 while for the mean minimum distance of (S_p, S_n) values from the genes between *E. coli* and *R. fascians* is 0.0021, which demonstrates that as indicated by BLAST, the genes of *R. fascians* are more distant than the genes of the *S. dysenteriae* in comparison with the genes of the *E. coli*.

4.2 Comparison of the Markov model of *E. coli* with genes of *Saccharomyces cerevisiae*

The Markov model can not only successfully identifies genetic similarities between different bacteria and predicts transition of group of words with similar efficiency, but can also be used to predict clustering in higher organism like *Saccharomyces cerevisiae* (baker's yeast) (genes of chromosome 1 were used for the analysis). Fig. 9(a) shows in red crosses the S_n and S_p values of genes of the *E.coli* and in blue empty squares the values for the genes of chromosome 1 of yeast. Therefore to create the (S_n, S_P) plot we define following terms $TP^{S.cer}(g_i, t^*) = \sum_{ij}^{1,1}(G_{ij}^{E.coli}(t^*) - G_{ij}^{S.cer}(g_i, t^*))$, where we only take into consideration all the i and j values of $G_{ij}^{E.coli}(t^*)$ and $G_{ij}^{S.cer}(g_i, t^*)$ which are equal to 1. The symbol $\sum_{ij}^{1,1}$ represents a summation that is only carried out when the variables inside the argument are equal to the super index. FN is the number of words that were wrongly predicted, $FN^{S.cer}(g_i, t^*) = \sum_{ij}^{1,0}(G_{ij}^{E.coli}(t^*) - G_{ij}^{S.cer}(g_i, t^*))$, this can happen only when $G_{ij}^{E.coli}(t^*) = 1$ and $G_{ij}^{S.cer}(g_i, t^*) = 0$, meaning that a transition from the group of words in box i are mapped to box j are not present but have been wrongly predicted by the model. $TN^{S.cer}(g_i, t^*) = \sum_{ij}^{0,0}(G_{ij}^{E.coli}(t^*) - G_{ij}^{S.cer}(g_i, t^*))$, in this case we consider all the values of $G_{ij}^{E.coli}(t^*)$ and $G_{ij}^{S.cer}(g_i, t^*)$ that are equal to zero, meaning that the a transition from the group of words in box i are mapped to box j do not exist and the model also does not predicts them. $FP^{S.cer}(g_i, t^*) = \sum_{ij}^{0,1}(G_{ij}^{E.coli}(t^*) - G_{ij}^{S.cer}(g_i, t^*))$, happens when $G_{ij}^{E.coli}(t^*) = 0$ but $G_{ij}^{S.cer}(g_i, t^*) = 1$. ($S_n$, S_p) values for genes of both organisms are clearly distinguishable, something to be expected since these two organisms have different genes. However, there are a few similar genes.

An amplification of Fig. 3(a) was done for determining any similar genes between *E. coli* and yeast shown in Fig. 3(a). The results shows few overlapping regions of S_p and S_n values between both organisms, indicating similarity between some genes. We analyse the similarity of these genes using BLAST2 which provided a coefficient of 0%, indicating no similarity. Our model therefore is capable of detecting similar patterns in genes that were before not correlated. Genes like ACS1 [6], GPB2 and FLC2 [7] involved mainly in histone acetylation, signalling regulations and cell-wall maintenance respectively of *Saccharomyces cerevisiae* were found closer to genes like lysR [8], lipA [9] and gcd [10] of *E. coli*, which are involved in activation elements, biosynthesis of lipoate and membrane bound protein respectively. Although the function of these genes of *E. coli* is not well known, our symbolic sequence analysis shows that these genes are too close to identified genes of yeast with well known functions.

4.3 Comparison of the Markov model of *E. coli* with a standard probabilistic model

Another standard way to model a genome is by constructing a probabilistic model (not a Markov model) of how words are mapped to other words, and not group of words. To create a model for words, which contains the same amount of transitions of our Markov model ($= 16^2$) we first start from the first sequence of the genome and slide a window to find all the possible words of length 4, 2 symbols in past (δ) and 2 symbols in future(γ). These words are encoded by a trajectory in the symbolic space (δ, γ). We construct a transition matrix $\mathbf{P}^*$ defined by transition probabilities of seeing a word encoded by the point (δ_i, γ_i) at a position in the DNA and then seeing the word encoded by the point (δ_j, γ_j), with j=i+1. The transition probability matrix $\mathbf{P}^{*E.coli}$ for the words generated for the genome of *E.coli* is calculated for the entire genome;

(a) **(b)**

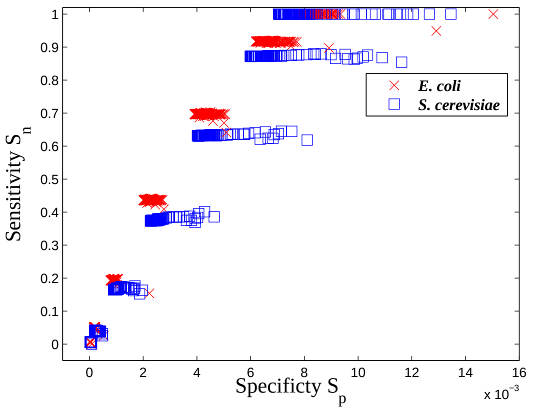

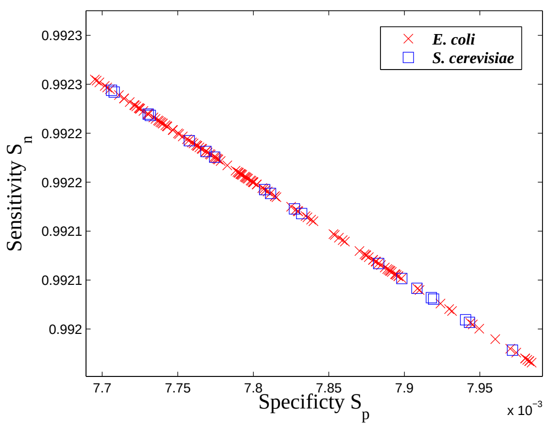

Figure 4: Comparative study done with *Saccharomyces cerevisiae* and Markov model of genes of E. coli, where red color cross represent genes of *E. coli* and blue colour boxes represent genes of yeast. (a) Prediction of genes of *Saccharomyces cerevisiae* (yeast) with Markov model for genes of *E.coli*. Horizontal axis shows Specificity S_p and vertical shows sensitivity S_n. From bottom to the top 7 different clusters represent results respectively from ($\tilde{N}_V = 5$, $t^* = 0.30$), ($\tilde{N}_V = 27$, $t^* = 0.27$), ($\tilde{N}_V = 85$, $t^* = 0.25$), ($\tilde{N}_V = 153$, $t^* = 0.23$), ($\tilde{N}_V = 207$, $t^* = 0.20$), ($\tilde{N}_V = 241$, $t^* = 0.15$) and ($\tilde{N}_V = 256$, $t^* = 0.0$). (b) An amplication for N=256, $t^* = 0$ for genes of *Saccharomyces cerevisiae* with the Markov model of *Escherchia coli*. Horizontal axis shows Specificity S_p and vertical shows sensitivity S_n.

we still divide the symbolic space in 256 boxes as we did it for our Markov Model. Then, we threshold the $\mathbf{P}^{*E.coli}(t^*)$ similarly as described in section 4 of the main paper. We generate an adjacency matrix $\mathbf{G}^{*E.coli}(t^*)$ from the transition probability matrix $\mathbf{P}^{*E.coli}(t^*)$ for different levels. Similarly, we create the transition matrix and adjacency matrix $\mathbf{P}^*(g_i, t*)$ and $\mathbf{G}^{*E.coli}(g_i, t^*)$ respectively for all the genes of *E. coli*. In order to compare our Markov Model with the probabilistic model we created we calculate the $(S_p(t^*),\ S_n(t^*))$ space for genes for *E. coli* as in Eqs. (18) and (19) of main paper, we re-define the parameters in these equations. For gene g_i, $TP^* = \sum_{ij}^{1,1}(G_{ij}^{*E.coli}(g_i, t^*) - G_{ij}(g_i, t^*))$, where we only take into consideration all the i and j values of $\mathbf{G}^{*E.coli}(g_i, t^*)$ and $\mathbf{G}(g_i, t^*)$ which are equal to 1. The symbol $\sum_{ij}^{1,1}$ represents a summation that is only carried out when the variables inside the argument are equal to the super index. FN is the number of words that were wrongly predicted, $FN^* = \sum_{ij}^{1,0}(G_{ij}^{*E.coli}(g_i, t^*) - G_{ij}(g_i, t^*))$, this can happen only when $G_{ij}^{*E.coli}(g_i, t^*) = 1$ and $G_{ij}(g_i, t^*) = 0$, meaning that a transition from the group of words in box i are mapped to box j are not present but have been wrongly predicted by the model. $TN^* = \sum_{ij}^{0,0}(Gij^{*E.coli}(g_i, t^*) - G_{ij}(g_i, t^*))$, in this case we consider all the values of $\mathbf{G}^{*E.coli}(t^*)$ and $\mathbf{G}(g_i, t^*)$ that are equal to zero, meaning that the a transition from the group of words in box i are mapped to box j do not exist and the model also does not predicts them. $FP^* = \sum_{ij}^{0,1}(G_{ij}^{*E.coli}(g_i, t^*) - G_{ij}(g_i, t^*))$, happens when $G_{ij}^{*E.coli}(t^*) = 0$ but $G_{ij}(g_i, t^*) = 1$.
Fig. 5 is a comparison between language models of *E.coli*. Our suggested Markov model i.e., (S_p, S_n) value for ($\mathbf{G}^{E.coli}$ and $\mathbf{G}(g_i)$) at different threshold t^* levels is shown in red cross and the (S_p, S_n) value for probabilistic model for $\mathbf{G}^{*E.coli}$ and $\mathbf{G}^*(g_i)$ is shown in blue colour empty boxes. As we can notice that at all t^* levels both the models have smallest S_p value and a large S_n value but for at $t^* = 0$, the predictability of genes by the group of words model (Markov model) is larger than the predictability by model for word transition (probabilistic model).
For most of the threshold t^* values, the Markov model has smallest specificity and larger sensitivity. However, specificity is at the same order for both models. The predominant difference is on the value of sensitivity which indicates that likelihood of doing a correct prediction is large in the

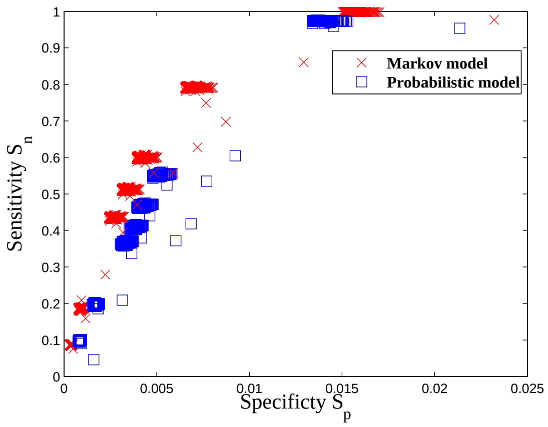

Figure 5: Specificity (S_p) and Sensitivity (S_n) curve for Markov model for group of words, represented by red crosses and probabilistic model of words represented by blue empty squares for genes of *E.coli*. From bottom to the top 7 different clusters represent results respectively from ($\tilde{N}_V = 5$, $t^* = 0.30$), ($\tilde{N}_V = 27$, $t^* = 0.27$), ($\tilde{N}_V = 85$, $t^* = 0.25$), ($\tilde{N}_V = 153$, $t^* = 0.23$), ($\tilde{N}_V = 207$, $t^* = 0.20$), ($\tilde{N}_V = 241$, $t^* = 0.15$) and ($\tilde{N}_V = 256$, $t^* = 0.0$).

Markovian model.

References

[1] Baptista M S, Maranhão D M, and Sartorelli J C. 2009 Dynamical estimates of chaotic systems from poincaré recurrences. *Chaos: An Interdisciplinary Journal of Nonlinear Science*, **19**, 043115. (doi: 10.1063/1.3263943)

[2] Bollt E, Góra P, Ostruszka A, and Życzkowski K. 2008 Basis markov partitions and transition matrices for stochastic systems. *SIAM Journal on Applied Dynamical Systems*, **7**, 341-360.

[3] Guanghong Z, Zhao X, and Bailin H. 2013 Shigella strains are not clones of escherichia coli but sister species in the genus escherichia. *Genomics, Proteomics and Bioinformatics*, **11**, 61 - 65.

[4] Altschul S F, Madden T L, Schaffer A A, Zhang J, Zhang Z, Miller W, and Lipman D J. 1997 Gapped blast and psi-blast: a new generation of protein database search programs. *Nucleic Acids Research*, **25**, 33893402.

[5] Hoeppner M, Latterner M, and Siyan K. 2013 *The NCBI Handbook [Internet]. 2nd edition.* Bethesda (MD): National Center for Biotechnology Information.

[6] Kratzer S and Schüller H-J. 1997 Transcriptional control of the yeast acetyl-coa synthetase gene, acs1, by the positive regulators cat8 and adr1 and the pleiotropic repressor ume6. *Molecular Microbiology*, **26**, 631-641.

[7] Byrne K P and Wolfe K H. 2005 The yeast gene order browser: Combining curated homology and syntenic context reveals gene fate in polyploid species. *Genome Research*, **15**, 1456-1461.

[8] Stragier P, Danos O, and Patte J-C. 1983 Regulation of diaminopimelate decarboxylase synthesis in escherichia coli: Ii. nucleotide sequence of the lysa gene and its regulatory region. *Journal of Molecular Biology*, **168**, 321-331.

[9] Reed K E and Cronan J E. 1993 Lipoic acid metabolism in escherichia coli: sequencing and functional characterization of the lipa and lipb genes. *Journal of Bacteriology*, **175**, 1325-1336.

[10] Matsushita K, Arents J C, Bader R, Yamada M, Adachi 0, and Postma P W. 1997 Escherichia coli is unable to produce pyrroloquinoline quinone (pqq). *Microbiology*, **143**, 3149-3156.

5 Appendix

N: Number of boxes
L: Length of symbolic sequence (past or future)
2L: Length of entire word (past and future)
$2L_n$: Length of word (past and future) that defines the name of each box
δ: Past symbolic sequence
γ: Future symbolic sequence
s: Position of nucleotide sequence in genome
τ: Iterations or time
$p(i)$: Probability of words of length 2L
H_T: Topological entropy
S_T: Shannon entropy
C: Correlation
T: defined as $C(\tau = T, N) \cong 0$
ϕ: Order of partition ($log_2(N)$)
MIR: Mutual Information Rate
$p_N(i)$: Probability of points being in box i in a partition with N^2 boxes
$p_N(j)$: Probability of points being in box j in a partition with N^2 boxes
$p_N(i|j)_\tau$: Transition probability of points being in box i going to box j after τ iterations
I_s: Mutual Information
$P_\delta(i)$: Marginal probabilities; Probability of points in column i
$P_\gamma(j)$: Marginal probabilities; Probability of points in row j
$P_{\delta\gamma}(i,j)$: Joint probability; Probability of finding points in the box (i,j)
$p(i)$: Probability of points being in box i
$p(j)$: Probability of points being in box j
$p(i|j)_1$: Transition probability of points being in box i going to box j in time=1
N_E: Number of edges for rescaled networks is denoted by $\tilde{N_E}$
N_V: Number of vertices and vertices for rescaled networks is denoted by $\tilde{N_V}$
$\mathbf{A}$: Transition matrix with elements (i,j) is denoted by $\mathbf{A}_{ij}$
$\mathbf{A}'$: Transition matrix with elements (i, j) with $\mathbf{A} > t^*$
$\tilde{\mathbf{A}}$: Rescaled transition matrix with elements (i,j) is denoted by $\tilde{A}_{ij}$
$\tilde{T}$: Time for which $\tilde{\mathbf{A}}$ has only non-null elements
E_d: Edge density
t^*: Range of threshold in network
M: Total measure of the system and for rescaled networks it is represented by $\tilde{M}$
$I_s(\tilde{N}_V, t^*)$: Mutual Information for rescaled network
$\tilde{p}(i)$: Probability of points in a rescaled matrix in column i
$\tilde{p}(j)$: Probability of points in a rescaled matrix in row j
$S(\tilde{N}_V, t^*)$: Shannon entropy for rescaled network
T_P: True Positives
F_N: False Negative
T_N: True Negative
F_P: False Positive
S_n: Sensitivity or True Positive Rate
S_p: Specificity or False Positive Rate
$\mathbf{G}^{E.coli}(t^*)$: Adjacency matrix constructed from transition matrix $\mathbf{A}(t^*)$ and rescaled transition matrix $\tilde{\mathbf{A}}(t^*)$ with elements (i,j) is denoted by $\mathbf{G}_{ij}^{E.coli}(t^*)$
g_i: Genes of *E.coli*
$\mathbf{B}(g_i, t^*)$: Transition matrix for each gene of the *E.coli* with elements (i,j) denoted as $B_{ij}(g_i, t^*)$
$\mathbf{G}(g_i, t^*)$: Adjacency matrix for each gene of *E.coli* with elements (i,j) denoted by $G_{ij}(g_i, t^*)$

$T_P^{E.coli}$: True Positives for each gene of *E.coli*
$F_P^{E.coli}$: False Positives for each gene of *E.coli*
$T_N^{E.coli}$: True Negatives for each gene of *E.coli*
$F_N^{E.coli}$: False Negatives for each gene of *E.coli*
$S_n^{E.coli}(t^*)$: Sensitivity for each gene of *E.coli* at different threshold levels (t^*)
$S_p^{E.coli}$: Specificity for each gene of *E.coli* at different threshold levels (t^*)
$\mathbf{G}^{S.dys}(g_i, t^*)$: Adjacency matrix for each gene of *S.dysenteriae* with elements (i,j) denoted by $G_{ij}^{S.dys}(g_i, t^*)$
$\mathbf{G}^{R.fas}(g_i, t^*)$: Adjacency matrix for each gene of $R.fascians$ with elements (i,j) denoted by $G_{ij}^{R.fas}(g_i, t^*)$
$T_P^{S.dys}$: True Positives for each gene of *S.dysenteriae*
$F_P^{S.dys}$: False Positives for each gene of *S.dysenteriae*
$T_N^{S.dys}$: True Negatives for each gene of *S.dysenteriae*
$F_N^{S.dys}$: False Negatives for each gene of *S.dysenteriae*
$T_P^{R.fas}$: True Positives for each gene of $R.fascians$
$F_P^{R.fas}$: False Positives for each gene of $R.fascians$
$T_N^{R.fas}$: True Negatives for each gene of $R.fascians$
$F_N^{R.fas}$: False Negatives for each gene of $R.fascians$
$T_P^{S.cer}$: True Positives for each gene of *S.cerevisiae*
$F_P^{S.cer}$: False Positives for each gene of *S.cerevisiae*
$T_N^{S.cer}$: True Negatives for each gene of *S.cerevisiae*
$F_N^{S.cer}$: False Negatives for each gene of *S.cerevisiae*
$\mathbf{P}^*$: Transition probability of seeing words encoded by a point
$\mathbf{P}^{*E.coli}(t^*)$: Transition probability of seeing words encoded by a point for *E.coli* at different threshold t^*
$\mathbf{G}^{*E.coli}(g_i, t^*)$: Adjacency matrix from the $\mathbf{P}^*$ for the standard probabilistic model
T_P^*: True Positives for each gene of *E.coli* from standard probabilistic model
F_P^*: False Positives for each gene of *E.coli* from standard probabilistic model
T_N^*: True Negatives for each gene of *E.coli* from standard probabilistic model
F_N^*: False Negatives for each gene of *E.coli* from standard probabilistic model
